# Editorial: COVID-19 pandemic: Mental health, life habit changes and social phenomena

**DOI:** 10.3389/fpsyt.2022.1105667

**Published:** 2022-12-15

**Authors:** Pedro Morgado, Xenia Gonda, Daria Smirnova, Konstantinos N. Fountoulakis

**Affiliations:** ^1^Life and Health Sciences Research Institute (ICVS), School of Health Sciences, University of Minho, Braga, Portugal; ^2^ICVS-3Bs PT Government Associate Laboratory, Braga/Guimarães, Portugal; ^3^Department of Psychiatry and Psychotherapy, Semmelweis University, Budapest, Hungary; ^4^NAP-2-SE New Antidepressant Target Research Group, Hungarian Brain Research Program, Semmelweis University, Budapest, Hungary; ^5^International Centre for Education and Research in Neuropsychiatry, Samara State Medical University, Samara, Russia; ^6^Third Department of Psychiatry, School of Medicine, Faculty of Health Sciences, Aristotle University of Thessaloniki, Thessaloniki, Greece

**Keywords:** anxiety, COVID-19 pandemic, depression, life style, mental health, public health, society, stress

The SARS-CoV-2 virus brought dramatic changes into daily life, subjecting society to the new and unforeseen era. The COVID-19 pandemic introduced challenges to governments, healthcare systems (including mental healthcare services), clinicians, and researchers worldwide, including management of healthcare sector investigations and international multicenter projects (1–5).

The COMET study was one of the largest quasi-epidemiological projects in the field of psychiatry which evaluated the impact of COVID-19 pandemic and its related lockdown conditions on the mental health of the 40 countries' population and was supported by the World Psychiatric Association. Study findings proved that pandemic was not just a threat to physical health but also presented severe stresses that broadly impacted the mental health and social lifestyles of people (6–9) (Panfil et al.). Its negative influence on the mental health of different vulnerable population groups has been described since the early beginning of the pandemic in 2020 (10–17).

This Research Topic was intended to describe the impact of COVID-19 pandemic on the population's mental health, life habits, daily beliefs, and social behaviors, as well as to discuss the urgent needs to face this evolving environment in the future. The 69 papers comprising this Research Topic, accepted from authors representing several countries and continents, examine the consequences of pandemic-associated factors investigated from multiple angles and points of view, and providing a really manifolded and detailed insight, not only broadening our understanding of the pandemic-related situation, the consequences of the lockdown conditions and similar crises, but also widening our knowledge in social, clinical psychiatry, and epidemiology of mental disorders.

COVID-19 has increased economic uncertainty, and not only negatively affected mental health, but also severely limited access to health services, which produced a cumulative burden in broad populations. The impact was differential and seemed to influence more significantly women (Batista et al.; Vrublevska et al.; Xie et al.; Alhazmi et al.; Bonzini et al.; Zhang et al.; Chutiyami et al.; Pisanu et al.; Eleftheriou et al.; Biswas et al.), younger people (Panfil et al.; Batista et al.; Chutiyami et al.; Pisanu et al.; Liu et al.), city inhabitants (Meyer et al.), and those persons who had experienced mental health problems in the past (Panfil et al.; Vrublevska et al.; Jang et al.; Ali et al.). Several studies identified depression (Meyer et al.; Jang et al.; Kim et al.) (18), anxiety (Vrublevska et al.; Alhazmi et al.; Folayan et al.; Fu et al.) (19), stress/distress (Krajewska-Kułak et al.; AlRasheed et al.) (19), burnout phenomenon (Chen, Bai, et al.) (20), post-traumatic stress disorder signs (Chutiyami et al.; De Pasquale et al.) (21), sleep disturbances (Folayan et al.; AlRasheed et al.), obsessive-compulsive symptoms (18, 22), and internet/mobile phone addiction (Jiang et al.; Moniri et al.) as the most common problems in the area of mental health observed in the general population.

The mental health of patients diagnosed with COVID-19 was also impacted by factors related to the pathophysiology of the SARS-CoV-2 infection and by various stressors multiplied during the quarantine period, and after release from quarantine. Anxiety and/or mood disturbances with psychomotor retardation as well as symptoms of impaired consciousness, memory, and insight were frequent and may be considered neuropsychiatric manifestations of COVID-19 (Sorokin et al.). Patients diagnosed with SARS-CoV-2 reported concerns about recovery and complications, stress related to social isolation measures, issues associated with the treatment environment, limited information about COVID-19 and infodemic, financial difficulties, stigma, discrimination, increased violence and conflicts within a family (Park et al.; Li et al.). Besides epidemiological findings, some of the presented papers describe background mechanisms which may also help to identify the targets for prevention and intervention in similar crisis situations.

During the pandemic, healthcare professionals were subject to extreme demands which pose significant short- and long-term effects on their mental health. Studies from several countries demonstrated the broad impact of the current pandemic on healthcare workers' mental health. A meta-review found that anxiety, depression, and stress/post-traumatic stress disorder were the most reported COVID-19 pandemic-related mental health conditions affecting healthcare workers (Chutiyami et al.). Other problems such as insomnia, burnout, fear, obsessive-compulsive disorder, somatization symptoms, phobia, cognitive failures, substance abuse, and suicidal thoughts were also reported (Chutiyami et al.; Mehri et al.). Those working in high-risk settings presented poorer mental health outcomes (Zhang et al.) (20).

Fortunately, not all that experience of stressful events related to the COVID-19 pandemic showed adverse consequences of it. In this vein, coping is defined as cognitive and behavioral efforts to deal with the demands of particular stressful situations minimizing their potential negative impacts. Physical exercises (Zhu et al.), yoga (Upadhyay et al.), and self-care activities (Gavurova et al.) within the daily routine were found beneficial. The most used coping or adjustment mechanisms were the avoidance-oriented coping with stress, emotion-oriented coping, and task-oriented coping (Twardowska-Staszek et al.). Interestingly, suppression has been shown as an adaptive response to the worry associated with uncertainty, at least, in the short-term context (Khatibi et al.). Among healthcare workers, the most-reported coping strategies include individual/group psychological support, family/relative support, training/orientation, and the adequacy of personal protective equipment (Chutiyami et al.).

The impact of the pandemic on society was significant but the ability to build effective responses was even more surprising. In a few months, a new and effective vaccine was developed and administered to millions worldwide significantly reducing the burden of the disease. Several diagnostic and therapeutic interventions were also developed both for COVID-19 symptoms and sequels as well as for its mental health consequences (Lee et al.; Asanjarani et al.; Hoseinzadeh et al.; Guelmami et al.; Schröder et al.).

As the knowledge of the virus increased and the correct information spread, the adaptation to stress also improved (23). In the early phases of the pandemic, public adherence to public health measures was high (Law et al.) but the spread of rumors, fake news, and misinformation was a challenge to governments, health authorities, and scientific institutions (Chen, Rong et al.) (24). Vaccination was particularly affected by misinformation. However, receiving information concerning COVID-19 vaccination from healthcare workers and scientific experts was associated with greater vaccination acceptance and decreased stress concerning COVID-19 vaccination (Zheng et al.; Vasileva et al.; Maciaszek et al.). Indeed, those who got the vaccine presented lower levels of depressive symptoms during the second wave of the infection outbreak (Zheng et al.; Benedetti et al.).

COVID-19 pandemic represents a public health emergency that exposed the dire consequences of inequality, affecting more negatively those who were more vulnerable before and at the beginning of the pandemic. Thus, economic support played a relevant role in the reduction of the negative impact of the pandemic contributing to alleviating symptoms of depression and anxiety (Yao et al.).

Humanity has learned a lot from this (perhaps, not so much) unexpected experience. The time is now to identify how we can be more resilient to future challenges. Current challenging times request us to rethink and to act.

## Author contributions

PM wrote the first draft. All authors wrote and approved the final manuscript.
